# HOW LONELY AND ISOLATED ARE OLDER PEOPLE WITH DEMENTIA AND THEIR CARERS: A DYADIC ANALYSIS

**DOI:** 10.1093/geroni/igz038.154

**Published:** 2019-11-08

**Authors:** Christina Victor, Isla Rippon, Anthony Martyr, Fiona Mathews, Linda Clare

**Affiliations:** 1 Brunel University London, Uxbridge, Middlesex, United Kingdom; 2 Brunel University London, Uxbridge, England, United Kingdom; 3 University of Exeter, Exeter, England, United Kingdom; 4 Newcastle University, Newcastle, England, United Kingdom

## Abstract

Studies of loneliness and isolation have rarely explored is how these experiences are reported within couples or the wider households. The IDEAL study has collected details of loneliness, as measured by the de Jong Gierveld (DJG) scale (range 0-6) and a single-item self-report measure, and isolation, using the six-item Lubben social network scale (range 0-30) from both people with dementia and carers. Loneliness is classified into three groups: not lonely (score 0-2), moderately lonely (3-4) and severely lonely (5+) and isolation into two: not isolated (score of 13+) or isolated (12 or less). Of the 1547 people with dementia and 1283 carers interviewed at baseline we have 1089 dyads who provided complete data on loneliness and 1204 for social isolation. Loneliness ratings are congruent between 43.1% of dyads and for 67.8% for isolation highlighting the subjective evaluative nature of loneliness as compared with more objectively measured isolation.

